# Proceedings of 2014 Stillbirth Summit

**DOI:** 10.1186/1471-2393-15-S1-A1

**Published:** 2015-04-15

**Authors:** Lindsey Wimmer

**Affiliations:** 1 Star Legacy Foundation, Eden Prairie, Minnesota, USA

## 

Stillbirth is a devastating pregnancy outcome that occurs once in every 160 pregnancies in the United States, [1] with similar rates in other developed nations around the world. Despite advances in medical knowledge and technology, there have been slow trends in stillbirth reduction in most high income countries over the past two decades relative to other declines in infant mortality [2]. Contributing to this lack of progress is the minimal amount of research activities dedicated to stillbirth etiologies and prevention methods.

As a follow-up to Stillbirth Summit 2011, Star Legacy Foundation hosted Stillbirth Summit 2014 in Minneapolis, MN (USA) June 19-21, 2014. The goals of the summit were to support current and emerging stillbirth research, promote collaboration among interested parties, and to encourage dialogue regarding the complex factors affecting stillbirth incidence and care. In attendance were researchers, health care professionals, stillbirth advocates, and bereaved families. The following oral papers offer an overview of the research presented and discussed at this event.
